# Mosquito excreta: A sample type with many potential applications for the investigation of Ross River virus and West Nile virus ecology

**DOI:** 10.1371/journal.pntd.0006771

**Published:** 2018-08-31

**Authors:** Ana L. Ramírez, Sonja Hall-Mendelin, Stephen L. Doggett, Glen R. Hewitson, Jamie L. McMahon, Scott A. Ritchie, Andrew F. van den Hurk

**Affiliations:** 1 College of Public Health, Medical and Veterinary Sciences, James Cook University, Cairns, Queensland, Australia; 2 Australian Institute of Tropical Health and Medicine, James Cook University, Cairns, Queensland, Australia; 3 Public Health Virology, Forensic and Scientific Services, Department of Health, Queensland, Australia; 4 Department of Medical Entomology, NSW Health Pathology-ICPMR, Westmead Hospital, Westmead, New South Wales, Australia; North Carolina State University, UNITED STATES

## Abstract

**Background:**

Emerging and re-emerging arthropod-borne viruses (arboviruses) cause human and animal disease globally. Field and laboratory investigation of mosquito-borne arboviruses requires analysis of mosquito samples, either individually, in pools, or a body component, or secretion such as saliva. We assessed the applicability of mosquito excreta as a sample type that could be utilized during studies of Ross River and West Nile viruses, which could be applied to the study of other arboviruses.

**Methodology/Principal findings:**

Mosquitoes were fed separate blood meals spiked with Ross River virus and West Nile virus. Excreta was collected daily by swabbing the bottom of containers containing batches and individual mosquitoes at different time points. The samples were analyzed by real-time RT-PCR or cell culture enzyme immunoassay. Viral RNA in excreta from batches of mosquitoes was detected continuously from day 2 to day 15 post feeding. Viral RNA was detected in excreta from at least one individual mosquito at all timepoints, with 64% and 27% of samples positive for RRV and WNV, respectively. Excretion of viral RNA was correlated with viral dissemination in the mosquito. The proportion of positive excreta samples was higher than the proportion of positive saliva samples, suggesting that excreta offers an attractive sample for analysis and could be used as an indicator of potential transmission. Importantly, only low levels of infectious virus were detected by cell culture, suggesting a relatively low risk to personnel handling mosquito excreta.

**Conclusions/Significance:**

Mosquito excreta is easily collected and provides a simple and efficient method for assessing viral dissemination, with applications ranging from vector competence experiments to complementing sugar-based arbovirus surveillance in the field, or potentially as a sample system for virus discovery.

## Author summary

Testing for the presence of arboviruses in mosquitoes used in laboratory experiments or surveillance usually involves collecting samples, from pools of hundreds of mosquitoes to the legs and wings of an individual mosquito and testing them by different methods. These methods can be labour intensive and costly and require sacrificing the mosquitoes. Arbovirus detection can be made from mosquito saliva; however, the amount of saliva mosquitoes expel is very small, making detection difficult. Here we demonstrate that mosquitoes excrete Ross River and West Nile viruses at levels sufficient to be detected by molecular assays as early as 2 days after they have fed on an infected blood meal. The amount of live (infectious) virus in excreta is low, suggesting that mosquito excreta poses a relatively low risk to people handling the samples. Mosquito excreta is easily collected in the laboratory and has a range of applications including experiments designed to incriminate mosquito species as vectors (i.e. vector competence experiments), arbovirus surveillance in the field, and discovery of previously unknown viruses.

## Introduction

It has been estimated that vector-borne diseases account for almost 20% of the global burden of infectious diseases, with more than 80% of the world’s population living in areas at risk [1]. Mosquitoes are the most important vectors of arthropod-borne viruses (arboviruses) globally. In recent years, many arboviruses have emerged or re-emerged due to several factors. High viral mutation frequency, widespread urbanization, and changes in land use, together with globalization and the growth of air travel, facilitate vector population increase and dispersal, and enable rapid transit of viremic humans [2, 3, 4]. Since few vaccines and antiviral therapies are available, critical work to understand and prevent arbovirus outbreaks must be undertaken both in the laboratory, by performing vector competence experiments to incriminate candidate species, and in the field by undertaking studies of virus ecology, as well as routine surveillance to identify periods of elevated virus activity.

Vector competence refers to the ability of a mosquito or other hematophagous arthropod to acquire, replicate, and successfully transmit a pathogen [5]. This is a key parameter to estimate vectorial capacity, namely the potential of a mosquito population to transmit an infectious agent to a susceptible host population [6]. Vector competence is determined by intrinsic factors that regulate virus infection of the midgut, escape from the midgut into the hemocel and associated tissues (dissemination), and finally infection of the salivary glands [7]. In the laboratory, vector competence is evaluated usually by feeding mosquitoes an infectious bloodmeal or allowing them to feed on an infected vertebrate. After a period of time, their ability to transmit the pathogen is evaluated. Several methods are used to assess transmission in the laboratory. Historically, transmission was evaluated by allowing mosquitoes to feed on susceptible vertebrate hosts (such as suckling mice) and then assessing infection (e.g. via clinical changes in the mice) [8, 9]. However, many arboviruses lack an appropriate model vertebrate host that will produce sufficient viremia or antibodies after exposure to be detected using standard laboratory assays [10]. Additionally, not all laboratories have the required biological security to allow handling vertebrate hosts in the same space as mosquitoes. Transmission can also be assessed *in vitro*, by forcing mosquitoes to salivate into capillary tubes [11] and then testing the expectorate for virus by inoculation in cell culture or by molecular assays. This method is relatively simple and removes ethical and logistical issues with working with live vertebrates. However, it can be an insensitive system to demonstrate transmission for some arboviruses, such as dengue viruses (DENVs) and chikungunya (CHIKV) [12,13]. Although not ideal, an alternative to estimate transmission potential is to test mosquito legs, wings, and/or heads, and use dissemination as a proxy for transmission [14]. This method fails to take into account possible salivary gland barriers to transmission [7, 15] and may overestimate the true transmission rate. The main limitation of *in vitro* methods is that since the mosquitoes must be sacrificed, they provide an end-point measurement preventing longitudinal measurements from the same individual.

In the field, routine arbovirus surveillance is carried out to detect elevated viral activity in order to implement disease control measures. Different strategies can be used for arbovirus surveillance [16] and one of the most widespread methods is the collection, identification, pooling and testing of wild mosquitoes by molecular assays or virus isolation. However, mosquito-based surveillance is time consuming and requires a continuous cold-chain to preserve virus viability for downstream processing. To overcome these limitations, a mosquito-free surveillance system based on the detection of arboviruses in saliva of infected mosquitoes has recently been developed [17, 18]. Saliva is collected on honey-baited nucleic acid preservation cards (Flinders Associate Technologies, FTA), which inactivate the virus and preserve viral RNA. Viral RNA is then eluted from the cards and detected using standard molecular assays. Importantly, the RNA preserved on the FTA cards serves as a template for nucleotide sequencing allowing strain identification and genotyping. This system has been successfully incorporated into routine surveillance programmes in Australia and is generally effective, as evidenced by numerous detections of arboviruses from multiple locations [19, 20, 21, 22]. Similar approaches using honey-baited cards or sugar-baited wicks have been evaluated in Florida [23] and California [24, 25]. Like any novel or emerging technology, there is always an opportunity to enhance the sugar-based arbovirus surveillance system. Since only a limited number of virions are passed during salivation [26, 27], the amount of virus on the FTA cards is generally of low concentration, indicating that the diagnostic assays are operating at their limits of detection [22]. This may lead to false negatives or insufficient template for downstream nucleotide sequencing. Additionally, this method will only detect mosquitoes after the extrinsic incubation period (EIP) which can take up to 14 days for some arboviruses. Finally, infection rates and vector species identification cannot be determined from honey-baited cards [28].

An exciting new application involves the collection of a previously overlooked sample. It was recently demonstrated by Fontaine et al. [29] that DENV RNA can be detected in excreta from *Aedes aegypti* mosquitoes with a disseminated infection. Since collection of excreta does not require sacrificing the mosquito, it allows for “time-to-event” estimation of the time for dissemination, and consequently, an estimation of the EIP when used as a proxy for transmission potential, in individual mosquitoes. Detection of viral RNA in mosquito excreta can also be used to select mosquitoes based on extreme phenotypes (viral refractory or susceptible) for experiments exploring the genetic basis of a complex trait. Mosquito excreta can potentially be used to complement sugar-based surveillance. Indeed, it appears that viral RNA detection in excreta is more sensitive than detection in saliva (89% vs 33% for DENV) [29]. Detection of arboviruses from excreta of infected mosquitoes could enable more sensitive detection of arboviruses than existing honey-baited FTA cards relying on collection of mosquito saliva alone.

The main objective of the current study was to determine whether mosquitoes excrete the Australian endemic arboviruses Ross River virus (RRV; family *Togaviridae*, genus *Alphavirus*,) and West Nile virus (Kunjin strain, WNV_KUN_; family *Flaviviridae*, genus *Flavivirus*) at levels sufficient to be detected by real-time reverse transcription polymerase chain reaction (RT-PCR) molecular assays. Building upon the Fontaine et al. [29] findings, we also determined if the association between virus dissemination and excretion extends to other arboviruses. Then, as a way to potentially enhance the sensitivity of the sugar-based surveillance system, we compared the detection of RRV and WNV_KUN_ in mosquito excreta with virus detected in saliva via filter paper cards. Importantly, in the context of workplace health and safety regulations affiliated with arbovirus surveillance systems, we evaluated whether excreted virus was infectious.

## Materials and methods

### Viruses

RRV was isolated from a pool of *Verrallina carmenti* collected from the Cairns suburb of Yorkeys Knob, Queensland, Australia in 2007 [30]. The virus had been previously passaged three times in African green monkey kidney (Vero) cells (ATCC, CCL-81). WNV_KUN_ was isolated from a pool of *Culex annulirostris* collected in the Gulf Plains region of Queensland, Australia in 2002 [31]. The virus had been previously passaged twice in porcine-stable equine kidney (PSEK) cells [32] before a final passage in *Aedes albopictus* (C6/36) cells (ATCC, CRL-1660).

### Mosquitoes

*Aedes vigilax* was selected based on its status as the coastal vector of RRV in Australia [33]. Eggs from colonized *Ae*. *vigilax* were obtained from NSW Health Pathology-ICPMR, Westmead Hospital, Westmead, Australia. The colony was originally established at the Malaria Research Unit at Ingleburn in 1986 from material collected near Townsville, Queensland. Eggs were hatched in 2L of 33% seawater containing ~45 mg of brain-heart infusion powder. Larvae were reared at 26°C 12:12 L:D and fed fish flakes (Tropical Flakes, Aqua One®, Ingleburn, Australia). Pupae were placed in 150 mL containers inside a 30 x 30 x 30 cm insect rearing cage. Emerged adults were held at 26°C, 75% RH and 12:12 L:D, and maintained on 15% honey solution *ad libitum*.

*Culex annulirostris* was selected based on its status as the primary WNV_KUN_ vector in Australia [34]. Adult mosquitoes were collected in February 2017 using passive box traps [35] baited with CO_2_ (1kg dry ice) and operated for 14 h (1700–0700) in a mixed *Melaleuca* and mangrove swamp near Cairns, Australia (−16.826613°, 145.707065°). These field mosquitoes were transported to the laboratory where they were briefly anesthetized and female *Cx*. *annulirostris* were sorted and maintained on 15% honey solution *ad libitum* at 26°C, 75% RH and 12:12 L:D. Since there is no evidence that WNV_KUN_ circulates in the Cairns region [30], it is unlikely that the mosquitoes had acquired the virus in the field.

### Virus exposure

Mosquitoes were starved for 24 h before oral infection with virus. Five to 7 day-old female *Ae*. *vigilax* were offered RRV diluted in washed defibrinated sheep blood (Institute of Medical and Veterinary Science, Adelaide, Australia) at 37°C using a Hemotek membrane feeding system (Discovery Workshops, Accrington, Lancashire, UK) with pig intestine as a membrane. *Cx. annulirostris* were exposed to WNV_KUN_ diluted in washed defibrinated sheep blood via the hanging drop method [36]. To determine the virus titer of the blood at the time of feeding and to assess if there was any reduction in titer, a 100 μL sample of the blood/virus mixture was taken before and after feeding, diluted in 900 μL of growth media (GM; Opti-MEM (Gibco, Invitrogen Corporation, Grand Island, NY) containing 3% foetal bovine serum (FBS; *In Vitro* Technologies, Australian origin), antibiotics and antimycotics), and stored at -80°C. After feeding, mosquitoes were briefly anesthetized with CO_2_ gas, and blood-engorged females sorted and placed in modified containers (see below) or in 900 mL containers covered with 100% polyester gauze (Spotlight Pty Ltd, Australia). All mosquitoes were maintained at 28°C, 75% RH and 12:12 L:D within an environmental growth cabinet for 15 days.

### Collection of excreta from mosquito batches

For each virus, 20 batches of 5 mosquitoes were placed in modified 200 mL polypropylene containers for excreta collection. The gauze-covered containers had a false floor made of fiberglass insect screen that allowed excreta to pass through onto a parafilm M (Bemis NA, Neenah, WI) disc situated about 5 mm below the screen to avoid cross contamination. Mosquitoes were fed on cotton balls soaked in 15% honey dyed with blue food colouring to allow for excreta visualisation and were replaced daily. Excreta was collected daily from day 2 to day 15 post-exposure (PE) using a cotton swab (Livingstone International, Rosebery, Australia) moistened with GM + 3% FBS. Each swab was placed in a 2 mL tube containing 1 mL GM + 3% FBS and stored at -80°C. Parafilm discs were replaced daily to avoid cross contamination. Mosquito mortality was also recorded daily. To compare the sensitivity of detection of viral RNA in excreta with the sensitivity of detection in saliva expectorates, on day 14 PE, mosquitoes were allowed to feed on a 4 cm^2^ filter paper card (FP; low chamber filter paper, Bio-Rad Laboratories, California) soaked in 100% honey dyed with red food colouring. After 24 h, the FP cards were removed, placed in a 2mL tube containing 1 mL GM + 3% FBS and stored at -80°C.

### Collection of excreta from individual mosquitoes

At three different timepoints (RRV: 7, 10, 14 days PE; WNV_KUN_: 6, 11, and 14 days PE), 20 individual mosquitoes were placed into 70 mL containers modified with the same design as described above. A 1 cm^2^ FP card soaked in 100% blue honey was offered as a sugar source. The mosquitoes were allowed to feed on the cards for 18–24 h, after which the excreta and the cards were collected as described above.

### Assessment of infection, dissemination and transmission rates from mosquito cohorts

Because the mosquitoes used for the batches and individual analyses were derived from a cohort exposed to the same infectious blood meal, we assessed the infection, dissemination and transmission rates only from the experiments that used individual mosquitoes. Saliva was collected using the *in vitro* capillary tube method described by Aitken [11] from mosquitoes described above. Bodies and legs+wings were stored separately in a 2mL tube containing 1 mL GM + 3% FBS with a single 5 mm stainless steel bead to assess for infection and dissemination, respectively. Saliva expectorates were expelled into a 2mL tube containing 500 μL of GM + 3% FBS. All samples were stored at -80°C.

### Virus assays

The blood/virus mixtures were titrated as 10-fold dilutions in 96-well microtiter plates containing confluent C6/36 cell monolayers. Bodies and legs+wings were homogenized using a QIAGEN Tissue Lyser II (Qiagen, Hilden, Germany) for 3 minutes at 26 hz and centrifuged briefly at 14,000 g. Mosquito homogenates (bodies, legs+wings) and saliva expectorates collected using capillary tubes were filtered using a 0.2 μm membrane filter (Pall Corporation, Ann Arbor, MI). Filtered mosquito homogenates were inoculated in duplicate and filtered saliva expectorates were inoculated in quadruplicate onto confluent C6/36 monolayers in 96-well microtiter plates. To assess the viability of virus in excreta, 50 excreta samples collected from mosquito batches (10 samples, 5 time points) were homogenized and filtered as described above, and inoculated as neat (not diluted) and as 10-fold dilutions onto confluent C6/36 monolayers in 96-well microtiter plates. Plates were incubated at 28°C for 7 days before being fixed in PBS/20% acetone with 0.2% BSA and stored at -20°C. Virus infection in cells was assessed using a cell culture enzyme immunoassay (CC-EIA) using monoclonal antibodies: B10 for RRV and 4G2 for WNV_KUN_ [37] (provided by Roy Hall, University of Queensland, Australia).

Thawed excreta samples were homogenized in the Tissue Lyser II as describe above. Thawed FP cards were maintained on ice and briefly vortexed every 5 min for 20 min [17]. Viral RNA was extracted from the excreta supernatant and eluted FP cards with a QIAxtractor (Qiagen, Hilden, Germany) using the QIAmp One-For-All nucleic acid kit (Qiagen, Hilden, Germany) according to the manufacturer’s instructions. Viral RNA was detected using real-time TaqMan RT-PCR assays specific for RRV [38] and WNV [22] in a Rotor-Gene 6000 real-time PCR cycler (Qiagen, Australia). With each run, positive controls included an extraction control (bovine viral diarrhoeal virus, BVDV) and a positive virus control extracted from a virus stock with known titer. Negative controls included at least one negative extraction control and a no-template control (molecular grade water). For each sample, the threshold cycle number (C_t_) was determined; lower C_t_ values correspond to a greater amount of viral template. Any sample with a C_t_ value ≥40 was considered negative [39].

### Analysis

For all the samples titrated in the CC-EIA, 50% endpoints (tissue culture infectious dose_50_, TCID_50_) were calculated using the method of Reed-Muench [40] and expressed as TCID_50_/mL. The Mann-Whitney U test was used to determine if there was a difference between the C_t_ values observed for excreta samples from batches and individuals, and between excreta samples and saliva expectorates on FP cards. Fisher’s exact test was used to compare the difference in between detection of viral RNA in excreta and detection of virus by CC-EIA in legs+wings, as an indication of virus dissemination. Scatter plots, heat maps and all statistical analyses were performed using GraphPad Prism version 7.0c (GraphPad Software, La Jolla CA, www.graphpad.com).

## Results

### Infection, dissemination and transmission rates in mosquito cohorts

For RRV with *Ae*. *vigilax*, the mean (± SD) virus titer at the time of feeding was 10^8.1±0.1^TCID_50_/mL and the overall infection rate was 82% (Table 1). For WNV_KUN_ with *Cx*. *annulirostris*, the mean (± SD) virus titer at the time of feeding was 10^7.3±0.3^ TCID_50_/mL and the overall infection rate was 42% (Table 2). All *Ae*. *vigilax* with confirmed RRV midgut infection developed a disseminated infection. Transmission of RRV was first observed on day 8 PE when 9/19 mosquitoes expectorated the virus. Only 76% (19/25) of *Cx*. *annulirostris* with confirmed WNV_KUN_ midgut infection developed a disseminated infection. Transmission of WNV_KUN_ was first observed on day 12 PE when 3/20 mosquitoes expectorated the virus.

**Table 1:** Infection, dissemination and transmission rates in *Ae*. *vigilax* exposed to 10^8.1±0.1^TCID_50_/mL of RRV tested at different days post exposure (PE).

Day PE	Infection^a^			Dissemination^b^			Dissemination/ Infection^c^			Transmission^d^			Transmission/ Dissemination^e^		
	n	%	95%CI	n	%	95%CI	n	%	95%CI	n	%	95%CI	n	%	95%CI
8	19	79	56–92	19	79	56–92	15	100	76–100	19	47	27–68	15	60	36–80
11	19	89	67–98	19	89	67–98	17	100	78–100	19	32	15–54	17	35	17–59
15	17	76	52–91	17	76	52–91	13	100	73–100	17	29	13–53	13	38	18–65
Total	55	82	69–90	55	82	69–90	45	100	91–100	55	36	25–50	45	44	31–59

^a^Number of mosquitoes tested, percentage of mosquitoes containing virus in their bodies, 95% confidence intervals

^b^Number of mosquitoes tested, percentage of mosquitoes containing virus in their legs+wing, 95% confidence intervals

^c^Number of infected mosquitoes, percentage of infected mosquitoes containing virus in their legs+wings, 95%CI, percentage, 95% confidence intervals

^d^Number of mosquitoes tested, percentage of mosquitoes containing virus in their expectorate collected in capillary tubes, 95% confidence intervals

^e^Number of mosquitoes with disseminated infection, percentage of mosquitoes with disseminated infection containing virus in their expectorate collected in capillary tubes, 95% confidence intervals

**Table 2:** Infection, dissemination and transmission rates in *Cx*. *annulirostris* exposed to 10^7.3 ±0.3^TCID_50_/mL of WNV_KUN_ tested at different days post exposure (PE).

Day PE	Infection^a^			Dissemination^b^			Dissemination/ Infection^c^			Transmission^d^			Transmission/ Dissemination^e^		
	n	%	95%CI	n	%	95%CI	n	%	95%CI	n	%	95%CI	n	%	95%CI
7	20	40	22–61	20	15	4–37	8	38	13–70	20	0	0–19	3	0	0–62
12	20	45	26–66	20	40	22–61	9	89	54–100	20	15	4–37	8	38	13–70
15	19	42	23–64	19	42	23–64	8	100	63–100	19	26	11–49	8	63	30–87
Total	59	42	31–55	59	32	22–45	25	76	56–89	59	14	7–25	19	42	23–64

^a^Number of mosquitoes tested, percentage of mosquitoes containing virus in their bodies, 95% confidence intervals

^b^Number of mosquitoes tested, percentage of mosquitoes containing virus in their legs+wing, 95% confidence intervals

^c^Number of infected mosquitoes, percentage of infected mosquitoes containing virus in their legs+wings, 95%CI, percentage, 95% confidence intervals

^d^Number of mosquitoes tested, percentage of mosquitoes containing virus in their expectorate collected in capillary tubes, 95% confidence intervals

^e^Number of mosquitoes with disseminated infection, percentage of mosquitoes with disseminated infection containing virus in their expectorate collected in capillary tubes, 95% confidence intervals

### Detection of viral RNA in excreta from batches of mosquitoes

**Fig 1:**
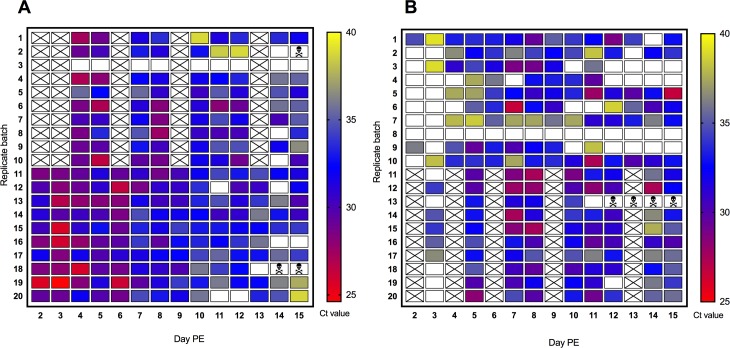
Real-time RT-PCR detection of arboviruses in excreta from 20 batches of 5 mosquitoes. (A) Detection of RRV RNA from *Ae*. *vigilax* excreta collected daily from day 2 to day 15 post exposure (PE) (B) Detection of WNV_KUN_ RNA from *Cx*. *annulirostris* excreta collected daily from day 2 to day 15 post exposure (PE). Lower C_t_ values correspond to a greater amount of viral template; a blank square indicates that viral RNA was not detected. A skull indicates that the container was removed from the experiment due to mortality of all 5 mosquitoes. X = not tested.

RRV and WNV_KUN_ viral RNA was excreted every day from day 2 PE onward in both *Ae*. *vigilax* and *Cx*. *annulirostris*, respectively, at levels sufficient to be detected by real-time RT-PCR. With the exception of one batch of *Ae*. *vigilax* and one batch of *Cx*. *annulirostris*, viral RNA was detected in excreta from all the batches of mosquitoes on at least one day (Fig 1). For RRV positive samples, C_t_ values ranged from 24.6 to 38.8. For WNV_KUN_ positive samples, C_t_ values ranged from 26.6 to 39.2.

### Detection of viral RNA in excreta from individual mosquitoes

**Fig 2:**
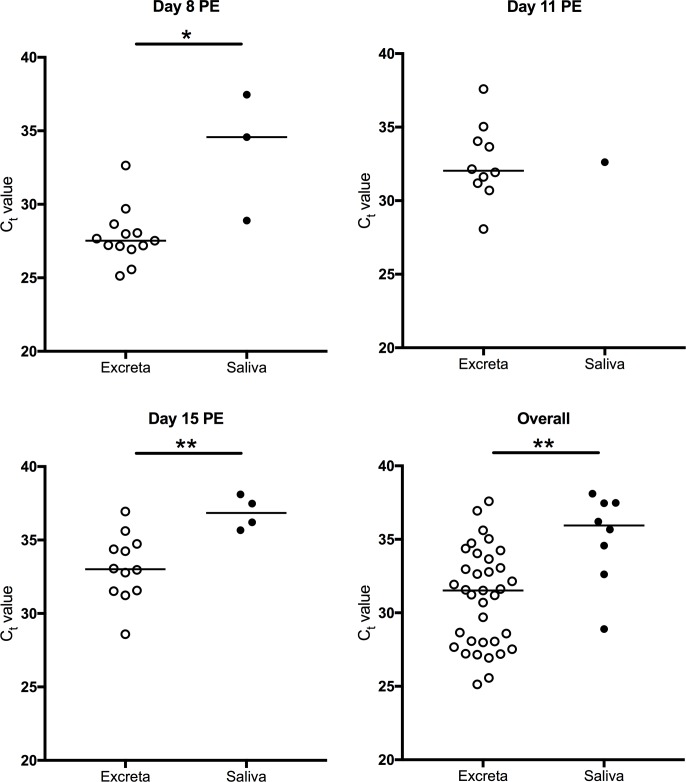
Detection of RRV RNA by real time RT-PCR in excreta swabs and saliva expectorates (filter paper cards). Samples collected over 18–24 h from individual *Ae*. *vigilax* sampled at different timepoints post exposure (PE). Bars denote medians. *P*<0.05 (*), *P*<0.001 (**), *P*<0.0001(***). Each point represents an individual mosquito.

It was possible to detect RRV RNA in excreta from individual *Ae*. *vigilax* on all days tested PE (Fig 2). Sixty-four percent (35/55) of samples were positive, with C_t_ values ranging from 25.1 to 37.6. No significant difference (*P*>0.05) was observed between the median C_t_ values from excreta collected from batches of mosquitoes and from individual mosquitoes, with the exception of day 8 PE where the median C_t_ value for batches was higher (30.8 vs 27.5; *P* = 0.0001, S1 Fig).

**Fig 3:**
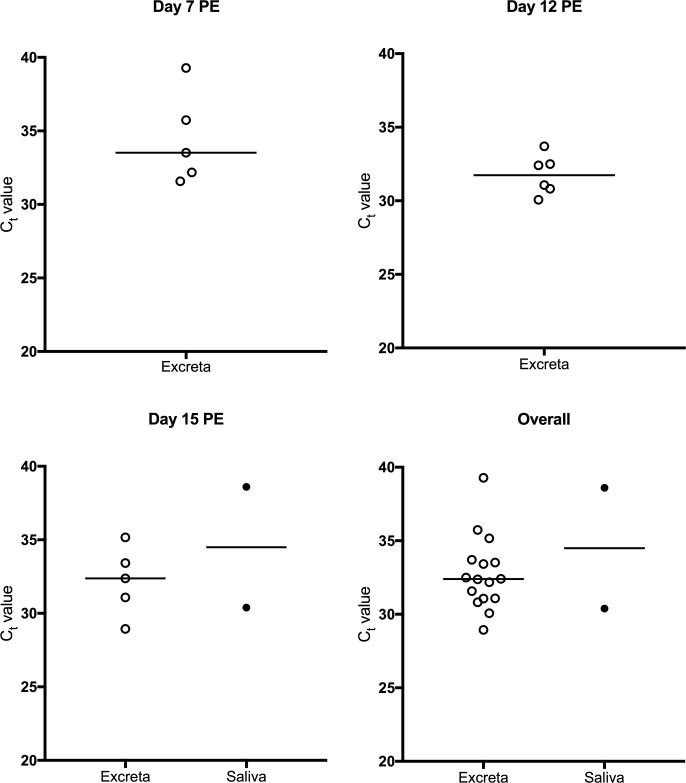
Detection of WNV_KUN_ RNA by real time RT-PCR in excreta swabs and saliva expectorates (filter paper cards). Samples collected over 18–24 h from individual *Cx*. *annulirostris* sampled at different timepoints post exposure. Bars denote medians. *P*<0.05 (*), *P*<0.001 (**), *P*<0.0001(***). Each point represents an individual mosquito. No mosquitoes expectorated virus onto filter paper cards on days 7 and 12 PE.

WNV_KUN_ RNA was detected in excreta samples from individual *Cx*. *annulirostris* tested on all days PE (Fig 3). Twenty-seven percent (16/59) of samples were positive, with C_t_ values ranging from 28.9 to 39.2. No significant difference (*P*>0.05) was observed between the median C_t_ values from excreta collected from batches of mosquitoes and from individual mosquitoes (S2 Fig).

### Association between disseminated infection and excretion of arboviruses

From 55 *Ae*. *vigilax* individuals tested, 45 (82%) mosquitoes had disseminated RRV infection. We detected RRV RNA in the excreta of 35 (78%) mosquitoes with a disseminated infection. None of the mosquitoes without a disseminated infection had positive excreta. From 59 *Cx*. *annulirostris* individuals tested, 19 (32%) had disseminated WNV_KUN_ infection. Thirteen (68%) mosquitoes with a disseminated infection had excreta positive for WNV_KUN_ RNA. Only 3 (8%) mosquitoes without disseminated infection had positive excreta. For both RRV and WNV_KUN_, there was a significant (*P*<0.0001) association between disseminated infection and excretion of viral RNA.

### Comparison of detection of arboviruses in excreta and saliva

**Fig 4:**
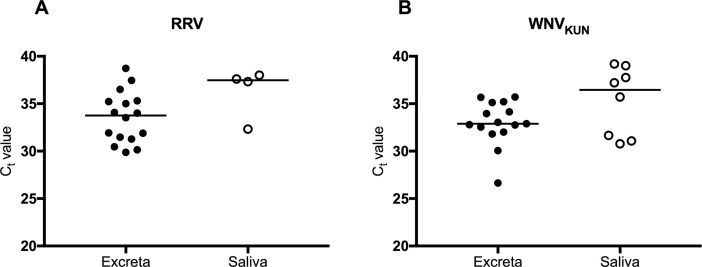
Detection of viral RNA in excreta and saliva expectorates (filter paper cards) from mosquito batches on day 15 post-exposure. (A) Detection of RRV RNA by real time RT-PCR in excreta and filter paper cards collected over 18–24 h from batches of 5 *Ae*. *vigilax*. (B) Detection of WNV_KUN_ RNA by RT-PCR in excreta and filter paper cards collected over 18–24 h from batches of 5 *Cx*. *annulirostris*.

Saliva deposited on FP cards from batches of mosquitoes on day 15 PE was tested for viral RNA. For *Ae*. *vigilax*, the proportion of RRV positive excreta samples was higher than the proportion of RRV positive FP cards (89% (16/18) vs 22% (4/18); *P*<0.0001). For *Cx*. *annulirostris*, the proportion of WNV_KUN_ positive excreta samples was higher than the proportion of WNV_KUN_ positive FP cards (79% (15/19) vs 42% (8/19); *P* = 0.0448). For both viruses, no significant difference (*P*>0.05) was observed between the median C_t_ values obtained from positive excreta and saliva expectorates on FP cards (Fig 4)

There was a significant difference (*P* < 0.05) between the proportions of RRV positive excreta and RRV positive FP cards obtained from individual *Ae*. *vigilax* at each time point (Table 3). With the exception of day 11 PE, where only one FP card was positive, median C_t_ values were significantly different between excreta and FP cards (day 8 PE: *P*<0.05; day 15 PE: P<0.01; overall: *P*<0.01; Fig 2).

**Table 3:** Proportion of excreta and saliva (filter paper cards) from individual mosquitoes positive for viral RNA by real-time RT-PCR tested at different days post exposure (PE).

Mosquito	Virus	Day PE	n	Excreta^a^		Saliva^b^	
				%	95%CI	%	95%CI
*Ae*. *vigilax*	RRV	8	19	68	46–85	16*	5–38
		11	19	53	32–73	5*	0–26
		15	17	71	47–87	24*	9–48
		Total	55	64	50–75	14*	7–26
*Cx*. *annulirostris*	WNV_KUN_	7	20	25	11–47	0*	0–19
		12	20	30	14–52	0*	0–19
		15	19	26	11–49	11	2–33
		Total	59	27	17–40	3*	3–12

^a^Percentage of positive excreta samples, 95% confidence intervals

^b^Percentage of positive saliva samples (filter paper cards), 95% confidence intervals

*Fisher’s exact test two-tailed *P*-value <0.05 for comparison with excreta

For WNV_KUN_ only 2 FP cards were positive on day 15 (Fig 3). With the exception of day 15 PE, there was a significant difference (*P* < 0.05) between the proportions of WNV_KUN_ positive excreta and FP cards obtained from *Cx*. *annulirostris* at different time points (Table 3). There was no significant difference (*P*>0.05) between median C_t_ values obtained from excreta and FP samples (Fig 3).

Specificity and sensitivity of viral RNA detection in excreta and FP cards as a proxy for viral dissemination were calculated as described by [29]. Mosquitoes with a confirmed disseminated infection (assessed by CC-EIA) and a positive RT-PCR result were considered true positives (TP) and those with a disseminated infection but a negative RT-PCR result were considered false negatives (FN). Mosquitoes without a disseminated infection and negative RT-PCR result were considered true negatives (TN) and those without a disseminated infection and positive RT-PCR result were considered false positives (FP). Using excreta as a proxy for viral dissemination, detection of RRV in excreta is highly specific (100%) and moderately sensitive (78%, 95%CI: 66–90). In contrast, detection of RRV in FP cards is highly specific (100%) but only slightly sensitive (18%, 95%CI: 7–29). For WNV_KUN_, detection in excreta also is highly specific (93%, 95%CI: 84–100) and moderately sensitive (68%, 95%CI: 48–90) while detection in FP cards is highly specific (100%) but slightly sensitive (11%, 95%CI: 0–24).

### Viability of arboviruses in excreta

To evaluate whether the excreted virus was infectious, 50 samples collected from batches of mosquitoes from each experiment (10 batches from 5 time points, RRV: day 2, 3, 6, 9 and 13 PE; WNV_KUN_: day 2, 4, 6, 9 and 13 PE) were inoculated onto C6/36 cells and virus infection confirmed using the CC-EIA. Only 3 samples (6%) from different batches on different days had sufficient material to quantify the amount of RRV (day 2PE: 10^3.06^ TCID_50_/mL; day 3PE: 10^1.30^ TCID_50_/mL; day 9PE: 10^1.80^TCID_50_/mL). Trace amounts of viable RRV were found on 8% (4/50) of the samples. In these samples CC-EIA indicated the presence of the virus in at least one well, but it was below the calculation cut-off value. Only one sample from day 9 PE showed trace amounts of viable WNV_KUN_ (2%, 1/50).

## Discussion

Our results confirm that mosquitoes exposed to RRV or WNV_KUN_ excrete viral RNA at levels sufficient to be detected by molecular assays. Our findings, together with previous observations on the excretion of DENV RNA by *Ae*. *aegypti* [29] support the hypothesis that the excretion of arboviruses by mosquitoes is a general phenomenon. Interestingly, even when the infection rate of WNV_KUN_ in *Cx*. *annulirostris* (42%) was lower than the infection rate of RRV in *Ae*. *vigilax* (82%), we were able to detect viral RNA in excreta from batches of mosquitoes continually from day 2 to day 15 PE. This indicates that the detection of viral RNA in excreta is not a result of a high mosquito infection rate under laboratory conditions. Blood meal digestion times vary between mosquito species, but generally 72 hours after feeding it has finalized [41]. Similar to the results of Fontaine et al., we observed brown excreta spots from digested blood meals in samples from day 2 and 3 PE, hence it is possible that viral RNA from those samples came directly from the blood meal. From day 4 onward, no dark excreta spots were visible, indicating that blood meal digestion was completed. The excreta from individual mosquitoes also provided sufficient material for detection of viral RNA at all timepoints indicating that the method is sensitive enough regardless of the volume of excreta collected. Indeed, we were able to detect viral RNA from containers with as little as one visible blue excreta spot.

We observed a correlation between viral dissemination and excretion of viral RNA. RRV RNA was not detected in excreta from any individual *Ae*. *vigilax* tested without a disseminated infection. Only 3 excreta samples from *Cx*. *annulirostris* without disseminated infection but with confirmed midgut infection were positive for WNV_KUN_ RNA. However, it is important to note that viral dissemination was assessed by cell culture, which is less sensitive than RT-PCR [42] and may have failed to detect low titer disseminated infection. RRV disseminates quickly in *Ae*. *vigilax*; 2 days after ingesting an infectious bloodmeal [33] with transmission occurring from day 3–4 PE [43]. Similarly, dissemination of WNV_KUN_ in *Cx*. *annulirostris* is detectable as early as day 3, with initial transmission observed on day 5 and increasing from day 10 to day 14 PE [44]. We detected RRV and WNV_KUN_ RNA in 90% and 70% excreta samples from batches of *Ae*. *vigilax* and *Cx*. *annulirostris*, respectively, collected on day 4 PE, when viral dissemination has already occurred for both viruses. Our results from individuals and batches of mosquitoes support the idea that testing mosquito excreta could be used in vector competence experiments as an indicator of viral dissemination or as a proxy for virus transmission potential for arboviruses that do not have a suitable transmission model, such as the DENVs, without having to sacrifice the insects. A limitation of this method is that it is impossible to distinguish viral RNA resulting from blood meal digestion from that being excreted because of viral dissemination. In order to avoid false positives, excreta samples should be collected after blood meal digestion has finalized.

For both batches and individual mosquitoes (overall), the proportion of positive excreta samples was higher than the proportion of positive saliva samples, suggesting that excreta offers an attractive sample for analysis of mosquitoes with disseminated infection in the laboratory and potentially in the field. Although specificity of detection of viral RNA when used as a proxy for viral dissemination in both excreta and saliva is high, sensitivity is at least 4 times higher for excreta compared to saliva (RRV: 78% vs 18%: WNV_KUN_: 68% vs 11%). Indeed, for WNV_KUN_ only 2 saliva samples were positive for viral RNA. These differences in sensitivity are expected, since detection of viral RNA in excreta and saliva result from different processes: dissemination and transmission. Not all mosquitoes with a disseminated infection transmit the virus, and the existence of a salivary gland infection barrier, where the virus is unable to enter or establish infection of the salivary glands prior to transmission has been documented. [7, 15]. In this experiment, only 44% and 42% of the mosquitoes with a disseminated infection transmitted RRV and WNV_KUN_, respectively, as measured by the capillary tube method. The median C_t_ values obtained from positive saliva expectorates were significantly higher than those from positive excreta samples obtained from individual mosquitoes. This is not surprising, since the volume of fluid excreted by mosquitoes is higher than what they expectorate (~1.5 μl [45] vs 4.7 nl [41]). This difference was not observed in batches of mosquitoes, possibly because there was more than one mosquito expectorating onto each filter paper card, potentially increasing the amount of viral RNA.

There is potential for mosquito excreta to be applied to enhance arbovirus surveillance. Honey-based surveillance provides a better estimate of transmission risk than testing pools of mosquitoes, since only transmitting mosquitoes will yield positive results [17, 46]. However, the proportion of mosquitoes in a population that survive the extrinsic incubation period can be low. Given that arboviruses can be detected in excreta as early as 2 days after the ingestion of an infectious blood meal, mosquito excreta could be used to obtain evidence of arbovirus circulation earlier. These results could be used to prompt intensive mosquito trapping for pooling and processing by traditional methods. Since mosquitoes expel only small quantities of saliva, the amount of virus on FTA cards is generally of low concentration which may lead to false negatives [22]. In this study, we observed that detection of arboviruses in excreta is more sensitive than detection in saliva. Further experiments will be required to establish if large amounts of excreta from non-infected mosquitoes would reduce the ability to detect viral RNA from the excreta of a single mosquito and to evaluate its performance under field conditions. Additionally, a methodology would need to be developed to collect and preserve the viral RNA from excreta in light traps and passive mosquito traps [18, 35] in a way that is convenient for routine surveillance. Recently, a method was described to collect mosquito excreta for xenomonitoring of filarial parasites, malaria, and trypanosomes, using super hydrophobic cones to concentrate excreta either into tubes or FTA cards, enabling detection of parasite DNA from the samples [47]. Finally, mosquito excreta could be used as an exploratory sample for virus discovery or metagenomic analysis by providing a template for next generation sequencing, greatly reducing associated costs (one sample vs several pools of mosquitoes per trap).

Only low or trace amounts of viable virus were found in excreta samples. It has been proposed that arbovirus virions in the midgut are inactivated by digestive proteases that affect the integrity of their envelope, rendering the virion non-infectious [7]. The sample with the highest titer (RRV, 10^3.06^ TCID_50_/mL) was obtained on day 2 PE and it is possible that this “higher” viral titer resulted from the digestion of the recently acquired infectious blood meal. It is unlikely that mosquito excreta has a role as an alternative route of transmission under field conditions. Firstly, arboviruses are labile in the environment; in fact, viability of arboviruses in infected mosquitoes decreases rapidly after their death in hot and humid conditions [48]. Mosquito excreta also contains digestive enzymes [49] which could continue to inactivate remaining virions once they have been excreted. Secondly, arbovirus infection via aerosol has only been observed under circumstances of high virus concentration [50]. Studies to test Japanese encephalitis virus (JEV) vaccines using *Rhesus* macaques exposed intranasally to JEV required at least 6.6 x 10^6^ infectious units per animal to achieve infection [51, 52]. Our results obtained from batches of 5 mosquitoes with a high infection rate showed only low or trace amounts of viable virus. In the field, where only 1–2 mosquitoes out of thousands in a trap might be infected, the amount of viable virus in excreta would be even lower. Finally, it is well documented that mosquito saliva plays an important role in facilitating arbovirus transmission [53] and excreta lacks salivary proteins responsible for generating favourable replication conditions in the vertebrate host.

There are some factors that influence the outcome of experiments that rely on experimental infection of mosquitoes. A limitation of our study was the use of field collected *Cx*. *annulirostris*. It has been documented that the source of the vector population plays a role in the outcome of vector competence studies [54]. Unknown factors such as age, previous exposure to other pathogens, temperature and vector microbiome can affect vector competence and the reproducibility of the experiment [55, 56]. Differences in blood meal titers could also influence rates of excreta detection. Midgut infection and escape barriers are dose dependent [57]. Females exposed to higher viral doses tend to develop a disseminated infection quicker. In contrast, females ingesting lower viral doses have lower infection rates and take longer to amplify the virus [58]. In our study, both mosquitoes were exposed to high viral titers, which could explain the early detection of viral RNA in excreta resulting from viral dissemination. While excreted viral RNA is detected earlier from mosquitoes exposed to higher titers, Fontaine et al. did not observe a difference in the amount of DENV RNA excreted between low and high titers. Further experiments will be required to determine if this applies to other arboviruses.

Important work to understand and prevent arbovirus outbreaks is undertaken in the laboratory and in the field analysing different mosquito samples. Mosquito excreta is an easily collected sample and provides a simple and efficient method for assessing virus dissemination in vector competence experiments. Although the use of mosquito excreta to enhance sugar-based arbovirus surveillance is still at experimental stage, our results suggest that excreta offers an attractive sample for analysis that could enable earlier and more sensitive detection of circulating arboviruses, and potentially be used for virus discovery.

## Supporting information

S1 Fig Detection of RRV RNA by real time RT-PCR in excreta from batches and individual mosquitoes. Samples collected over 18–24 h from batches and individual *Ae*. *vigilax* sampled at different timepoints post exposure (PE). Bars denote medians. *P*<0.05 (*), *P*<0.001 (**), *P*<0.0001(***). Each point represents either a batch of 5 or an individual mosquito.
(TIFF)

S2 Fig Detection of WNVKUN RNA by real time RT-PCR in excreta from batches and individual mosquitoes. Samples collected over 18–24 h from batches and individual *Cx*. *annulirostris* sampled at different timepoints post exposure (PE). Bars denote medians. *P*<0.05 (*), *P*<0.001 (**), *P*<0.0001(***). Each point represents either a batch of 5 or an individual mosquito.
(TIFF)
