# Supplementary figures and images for: Mosquito excreta: A sample type with many potential applications for the investigation of Ross River virus and West Nile virus ecology
Source: PLoS Negl Trop Dis. 2018 Aug 31;12(8):e0006771. doi: 10.1371/journal.pntd.0006771 (PMC6136815; doi:10.1371/journal.pntd.0006771)

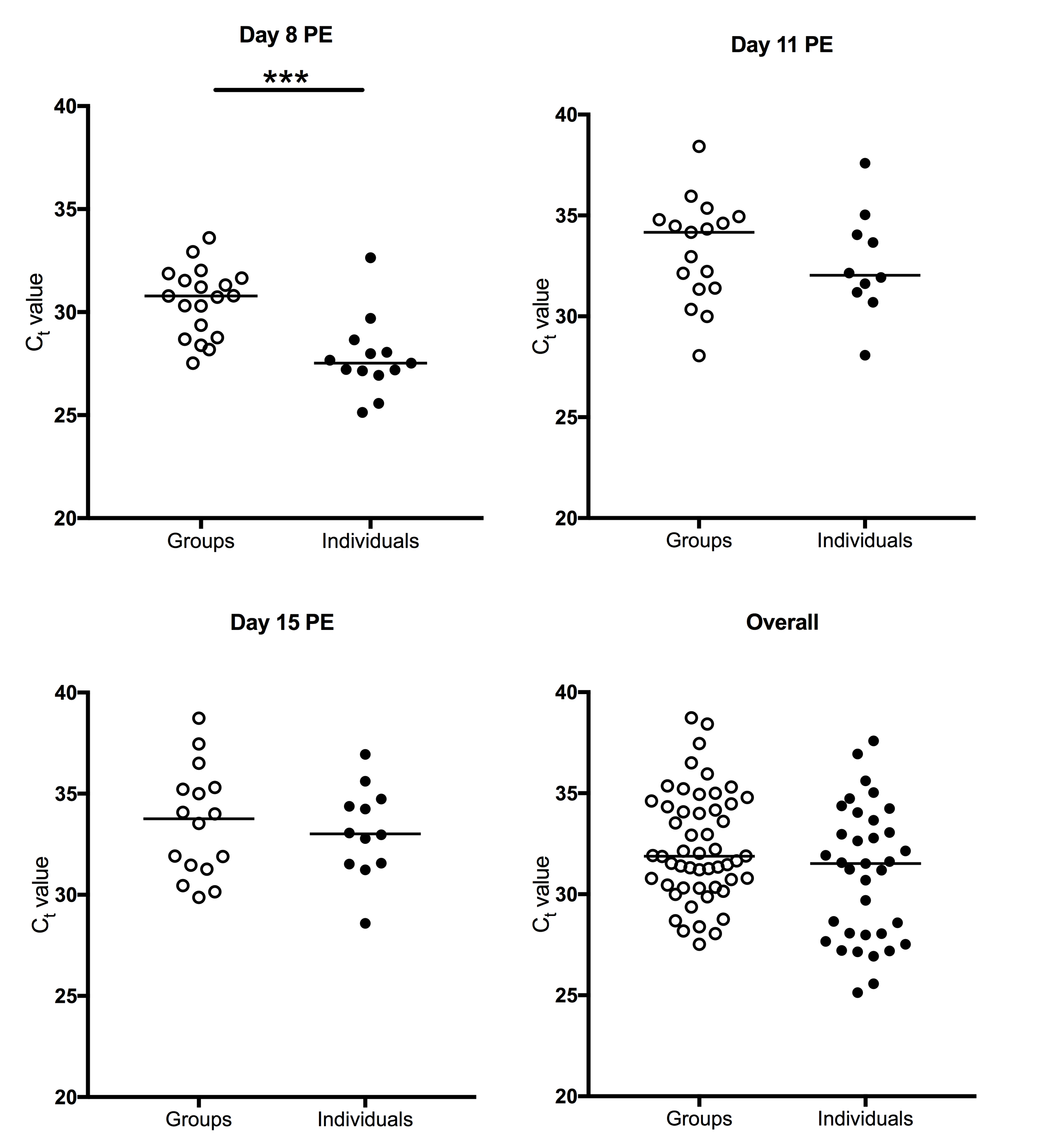

Supplement: S1 Fig — Samples collected over 18–24 h from batches and individual Ae. vigilax sampled at different timepoints post exposure (PE). Bars denote medians. P<0.05 (*), P<0.001 (**), P<0.0001(***). Each point represents either a batch of 5 or an individual mosquito. (TIFF) [file pntd.0006771.s001.tiff]

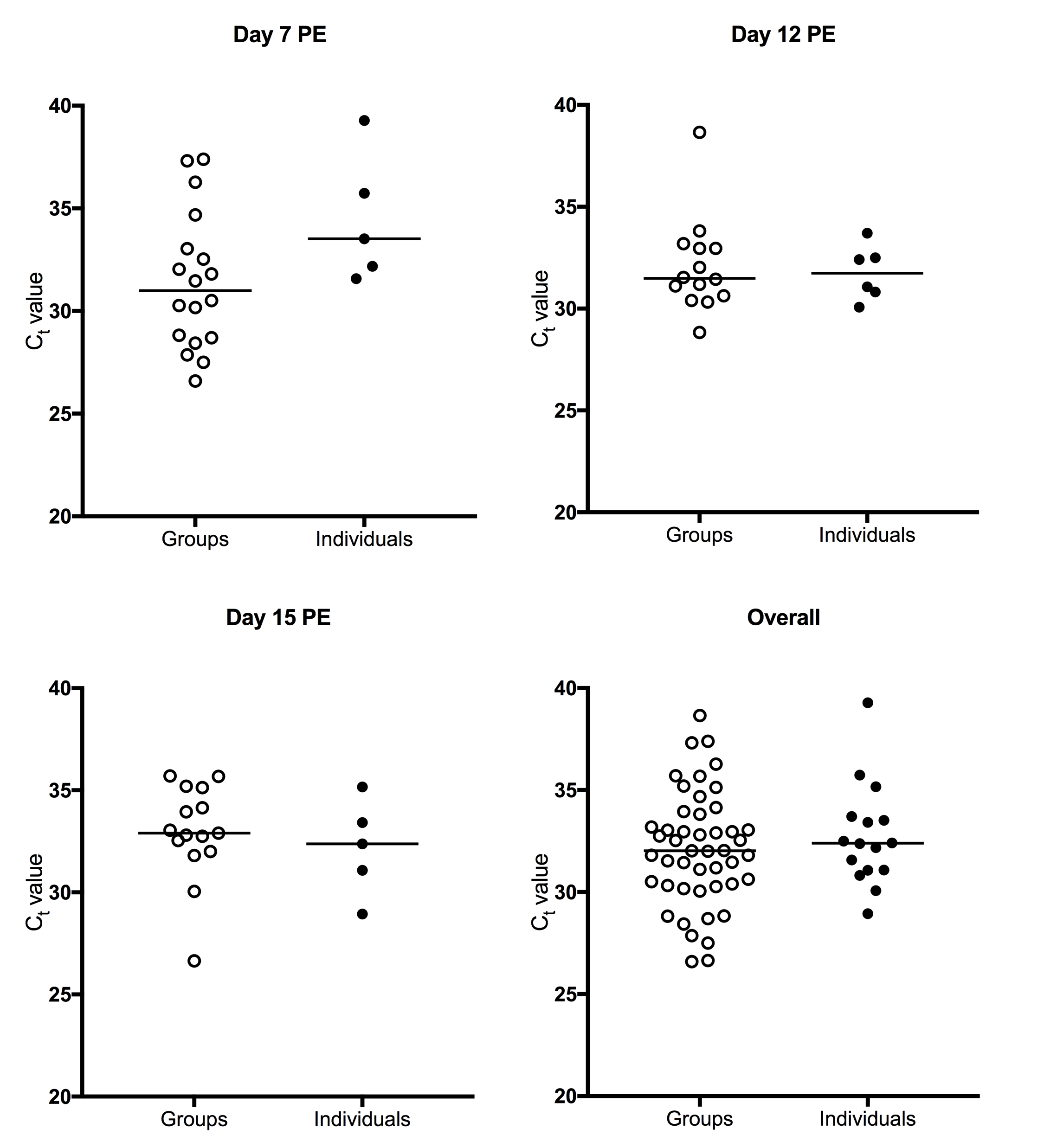

Supplement: S2 Fig — Samples collected over 18–24 h from batches and individual Cx. annulirostris sampled at different timepoints post exposure (PE). Bars denote medians. P<0.05 (*), P<0.001 (**), P<0.0001(***). Each point represents either a batch of 5 or an individual mosquito. (TIFF) [file pntd.0006771.s002.tiff]
